# Progress in Research on Bioactive Secondary Metabolites from Deep-Sea Derived Microorganisms

**DOI:** 10.3390/md18120614

**Published:** 2020-12-02

**Authors:** Ya-Nan Wang, Ling-Hong Meng, Bin-Gui Wang

**Affiliations:** 1 Key Laboratory of Experimental Marine Biology, Institute of Oceanology, Chinese Academy of Sciences, Nanhai Road 7, Qingdao 266071, China; wangyn@qdio.ac.cn; 2 Laboratory of Marine Biology and Biotechnology, Qingdao National Laboratory for Marine Science and Technology, Wenhai Road 1, Qingdao 266237, China; 3 College of Earth Science, University of Chinese Academy of Sciences, Yuquan Road 19A, Beijing 100049, China; 4 Center for Ocean Mega-Science, Chinese Academy of Sciences, Nanhai Road 7, Qingdao 266071, China

**Keywords:** deep-sea fungi, deep-sea actinomycetes, secondary metabolites, bioactivity

## Abstract

Deep sea has an extreme environment which leads to biodiversity of microorganisms and their unique physical and biochemical mechanisms. Deep-sea derived microorganisms are more likely to produce novel bioactive substances with special mechanism of action for drug discovery. This article reviews secondary metabolites with biological activities such as anti-tumor, anti-bacterial, anti-viral, and anti-inflammatory isolated from deep-sea fungi and bacteria during 2018–2020. Effective methods for screening and obtaining natural active compounds from deep-sea microorganisms are also summarized, including optimizing the culture conditions, using genome mining technology, biosynthesis and so on. The comprehensive application of these methods makes broader prospects for the development and application of deep sea microbial bioactive substances.

## 1. Introduction

Deep sea is one of the latest extreme environments developed on earth. The deep sea is an environment with extreme features including: (1) For every 10 m of increase in depth, the pressure increases by one atmosphere, so the water pressure is higher than 1000 atmospheres in the deep sea trench; (2) The temperature decreases with depth, which is usually around 2 °C on the deep sea bottom; (3) The seawater oxygen concentration mainly depends on the absorption of oxygen at the sea-air interface, the photosynthesis rate of autotrophs in the true light layer, and rate of consumption of marine life respiration; (4) The light intensity is close to zero below the depth of 250 m [1]. In conclusion, deep sea has the characteristics of extreme ecological environment, including high pressure, low temperature, lack of oxygen and darkness. The cold seeps, hydrothermal and seamounts of the world deep-sea locations may worth favoring for bioprospection. Deep-sea microbes have unique biological metabolic pathways to deal with extreme ecological environments, especially stress. Many deep-sea microbes are hypertrophic or pressure-sensitive. Existing research methods limit the cultivation of these steps [2].

Over the past fifty years, more than 30,000 marine natural products have been discovered, of which about 2% are derived from deep-sea microorganisms [3]. Based on our review of the literature, the number of marine natural products from deep-sea have increased since then, but they are still a small percentage of the total amount found. Also, we found that most recent researches on bioactive secondary metabolites are derived from bacteria and fungi in deep-sea environment. So, this review mainly covers natural products from deep-sea derived fungi and bacteria who were almost isolated from sediment or sea water. Among the natural products, people pay the most attention to compounds with antibacterial activity, especially for their application in the field of biotechnology and pharmaceuticals. The discovery of antibiotics with new structures is very important for dealing with the spread of resistant bacteria [4]. To a large extent, it is related to the isolation and cultivation of unknown deep-sea microorganisms and the discovery of related secondary metabolites. Although it is supposed that microorganisms are huge in number and rich in diversity in these environments [5], few have been characterized so far [6]. In fact, the discovery of deep-sea microbial diversity can lead to the discovery of compounds with new biological activities, further promoting the drug development process [7]. The first antibacterial natural product isolated from deep-sea sediments was a glial toxin produced by the metabolism of a fungus species, *Penicillium* sp., isolated from the Seto Inland Sea, Japan, which inhibits the growth of Gram-positive bacteria *Staphylococcus aureus* and *Bacillus subtilis* [8]. 

In the past three years, the development of deep-sea exploration and molecular biology provided technical support for the exploitation of deep-sea microbial natural products. To enable researchers to better understand the research work in these fields, this article summarized the characteristics of secondary metabolites isolated from deep-sea microorganisms and their biological activities in 2018–2020, as well as research methods for diversity of secondary metabolites from deep-sea microorganisms.

## 2. Secondary Metabolites from Deep-Sea Derived Fungi

Recent studies have shown that fungi from the extreme environments are potential producers for clinically important natural products [9] and may be the next frontier of drug discovery [10].

**Figure 1:**
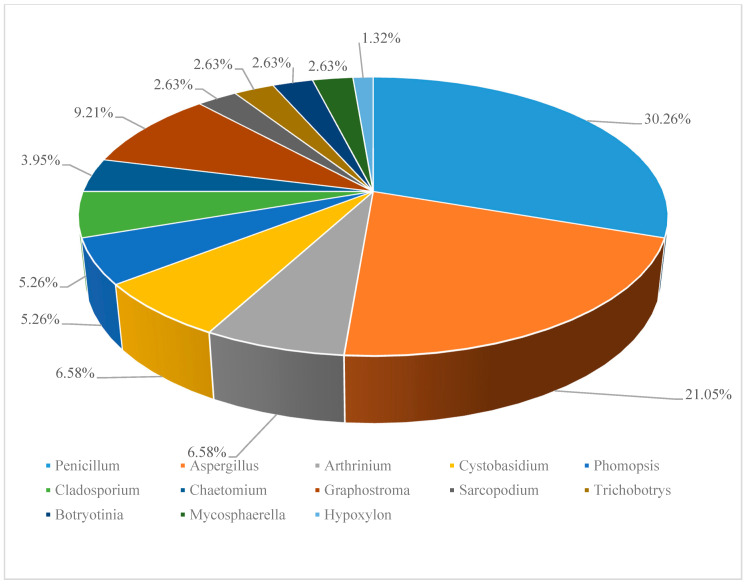
Distribution of deep-sea derived fungi in secondary metabolites discovery. (*Data based on the statistics of references [11,12,13,14,15,16,17,18,19,20,21,22,23,24,25,26,27,28,29,30,31,32,33,34,35,36,37,38]).

According to the references, 829 (194 novel) natural products were discovered from deep-sea derived fungi in the past three years, 79 among which showed biological activities (Table 1). Most of these compounds were isolated from species of two genera of fungi, *Penicillium* sp. (23, accounting for 30.26% of the total compounds) and *Aspergillus* sp. (16, accounting for 21.05% of the total compounds) (Figure 1). 

### 2.1. Antitumoral Secondary Metabolites

Compounds **1**–**32** were isolated from deep-sea fungi from different sea areas and showed varying degrees of antitumor activity.

**Figure 2:**
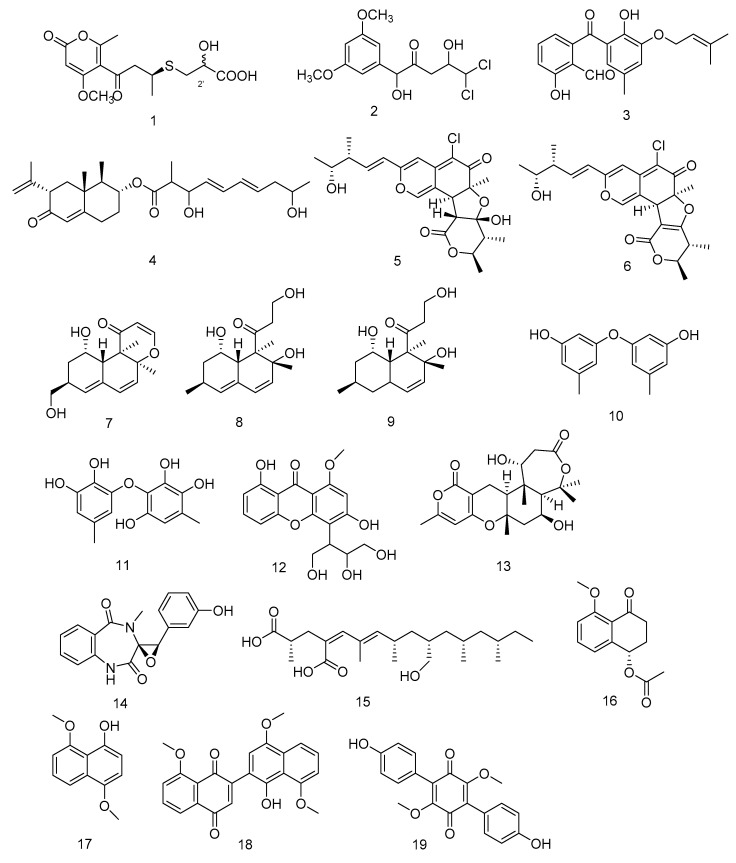
Structures of polyketides with antitumor activity.

Compounds **1**–**4** (Figure 2) are all polyketides. New compounds 2-hydroxyl-3pyrenocine-thio propanoic acid (**1**) and 5, 5-dichloro-1-(3, 5dimethoxyphenyl)-1, 4-dihydroxypentan-2-one (**2**), containing sulfur or chlorine atoms, were isolated from the ethyl acetate extract of *Penicillum citreonigrum*. Compound **1** existed in the form of C-2′ epimers with a ratio of 1:2 (2′*R*:2′*S*). It showed potent cytotoxicity against human hepatocellular carcinoma cell (HCC) line Bel7402 and human fibrosarcoma cell line HT1080. The IC_50_ (50% inhibiting concentration) values were 7.63 ± 1.46 and 10.22 ± 1.32 µM, respectively. Compound **2** exhibited an IC_50_ value of 16.53 ± 1.67 µM against human fibrosarcoma tumor cell HT1080 [11]. Benzophenone derivatives tenellone H (**3**) and compound AA03390 (**4**) were isolated from the extract of a fungus *Phomopsis lithocarpus* derived from Indian Ocean sediments, compound **3** exhibited moderate cytotoxic activity against human HCC line and human non-small cell lung cancer cell (NSCLC) line A549, with IC_50_ values of 16.0 ± 0.1 and 17.6 ± 0.3 µM, respectively. Compound **4** showed weak cytotoxic activity against human HCC cell line HepG-2, human breast cancer cell line MCF-7, human neuronal cancer cell line SF-268 and human NSCLC line A549 [12]. 

*Chaetomium globosum* HDN151398 was isolated from deep-sea sediments in the South China Sea, and its metabolites chaetomugilin A (**5**) and chaetomugilin C (**6**) (Figure 2) showed broad-spectrum cytotoxic activities. Compound **5** exhibited significant cytotoxic activity against human promyelocytic leukemia cell line HL-60 and human colorectal cancer cells HCT-116, with IC_50_ values of 6.4 and 6.1 µM, respectively. While compound **6** exhibited IC_50_ values of 6.6 and 5.7 µM against HL-60 and HCT-116, respectively [13].

Peniciversiols A (**7**), decumbenone A (**8**), decumbenone B (**9**), 3, 3′-dihydroxy-5, 5′-dimethyldiphenyl ether (**10**), violaceol-II (**11**), 3, 8-dihydroxy-4-(2, 3-dihydroxy-1-hydroxymethylpropyl)-1-methoxyxanthone (**12**), asperdemin (**13**), cyclopenol (**14**) and radiclonic acid (**15**)were isolated from the ethyl acetate extract of *Penicillium chrysogenum* MCCC 3A00292.Their structures were shown in Figure 2. Compound **7** was a versiol-type analogue featuring a 2, 3-dihydropyran-4-one ring and showed significant cytotoxic activity against human Bladder cancer cell line BIU-87 with the IC_50_ value of 10.21 μM. Meanwhile compounds **10**, **14** and **15** also had selective inhibition against BIU-87 with IC_50_ values of 16.41, 8.34 and 12.47 μM, respectively. Compounds **8**, **9**, **11** and **16** exhibited selective inhibitory effect against human esophageal cell line ECA109 with IC_50_ values of 12.41, 15.60, 8.95 and 7.70 μM, respectively. Compounds **12**–**15** had selective inhibition against human Hepatocellular carcinoma cell line BEL-7402. The IC_50_ values were 15.94, 12.75, 7.81 and 13.75, respectively [14].

*Hypoxylon rubiginosum* FS521, a higher fungi species, was isolated from deep-sea sediments in the South China Sea. From its ethyl acetate extract, hypoxone A (**16**), 4, 8-dimethoxy-1-naphthol (**17**), 1′-hydroxy-4′, 8, 8′-trimethoxy[2, 2′]binaphthalenyl-1, 4-dione (**18**) and 3, 6-dimethylatromentin (**19**) were isolated. Compounds **17** and **18** were new natural products. Compound **18** exhibited significant selective inhibitory effects against human glioblastoma carcinoma cell line SF-268, human breast cancer cell line MCF-7, human liver cancer cell line HepG-2 and human lung cancer cell line A549 with IC_50_ values of 1.9, 3.2, 2.5, and 5.0 μM, respectively. Compounds **16**, **17** and **19** showed weak inhibition against the four tumor cell lines with IC_50_ values ranging from 18.89 to 69.62 μM [15].

**Figure 3:**
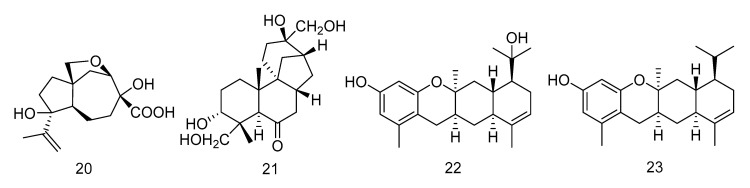
Structures of terpenoids with antitumor activity.

Penigrisacid D (**20**) is a sesquiterpene isolated from the extract of *Penicillium griseofulvum*, which showed a weak inhibitory activity against esophageal cancer cell line ECA-109, with an IC_50_ value of 28.7 µM [16]. Aphidicolin A8 (**21**) is a diterpene isolated from the extract of *Botryotinia fuckeliana* derived from Western Pacific seawater samples, which induced human bladder cancer cell T24 and human promyelocytic leukemia cell HL-60 apoptosis by DNA damage, with the IC_50_ values of 2.5 μM and 6.1 μM, respectively [17]. Photeroids A (**22**) and B (**23**) were isolated from the extract of the fungus *Phomopsis tersa*. They are both heteroterpenes containing a 6/6/6/6 tetracyclic system which forms Ortho-Quinone methides (o-QMs) intermediates through a rare Diels-Alder reaction. Compounds **22** and **23** (Figure 3) showed moderate cytotoxicity against four human cancer cell lines: SF-268, MCF-7, HepG-2 and A549 [18].

N-glutarylchaetoviridins (**24**) is an azaphilone alkaloid containing glutamine residues isolated from *Chaetomium globosum* HDN151398. Compound **24** exhibited significant cytotoxic activity against human gastric cancer cell line MGC-803 and human ovarian cancer cell line HO-8910, with IC_50_ values of 6.6 and 9.7 µM, respectively [13]. Apiosporamide (**25**) is also an alkaloid, isolated from the extract of *Arthrinium* sp. UJNMF0008 derived from sediments of the South China Sea. It exihibited cytotoxity against two human osteosarcoma cell lines (U2OS and MG63) with IC_50_ values of 19.3 and 11.7 µM [19].

**Figure 4:**
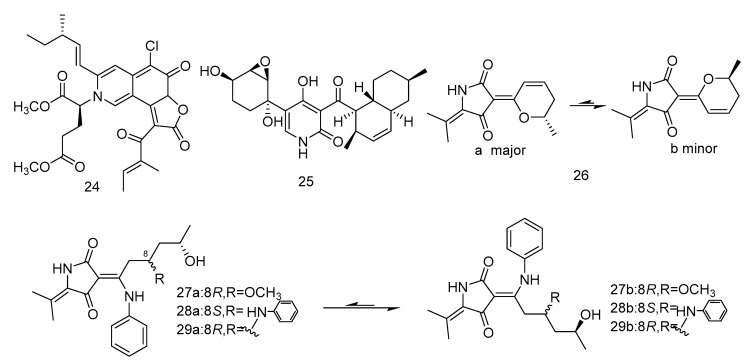
Structures of alkaloids with antitumor activity.

Cladodionen (**26**) was isolated from the extract of *Cladosporium sphaerospermum* derived from the Indian Ocean deep sea sediments, and cladosins I–K (**27**–**30**) (Figure 4) were isolated from the extract of *Cladosporium sphaerospermum* derived from Mariana Trench. Compound **26** showed cytotoxic activity against human promyelocytic leukemia cell line HL-60, with the IC_50_ value of 28.6 µM [20]. Compounds **27**–**29** showed different levels of cytotoxic activity against human chronic myelogenous leukemia cell line K562 and human promyelocytic leukemia cell line HL-60, with IC_50_ values ranging from 2.8 to 7.8 µM [21].

**Figure 5:**
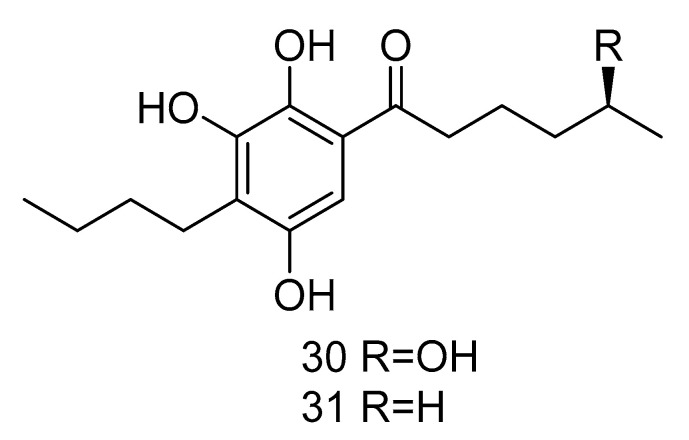
Structures of other compounds with antitumor activity.

Sarcopodinols A (**30**) and B (**31**) (Figure 5) were isolated from the fungus *Sarcopodium* sp. FKJ-0025 isolated from coastal sediments of Kagoshima. Compound **30** had weak cytotoxity towards human T-lymphocytic leukemia Jurkat cell line, with the IC_50_ value of 47 μg/mL. Compound **31** exhibited an IC_50_ value of 37 μg/mL against HL-60 cell line, 47 μg/mL against Jurkat cell line, and 66 μg/mL against human pancreatic cancer Panc1 cell [22].

### 2.2. Antmicrobial Secondary Metabolites

#### 2.2.1. Antibacterial Secondary Metabolites

Compounds **32**–**62** are secondary metabolites with antibacterial activity isolated from fungi extracts from different deep-sea environments.

**Figure 6:**
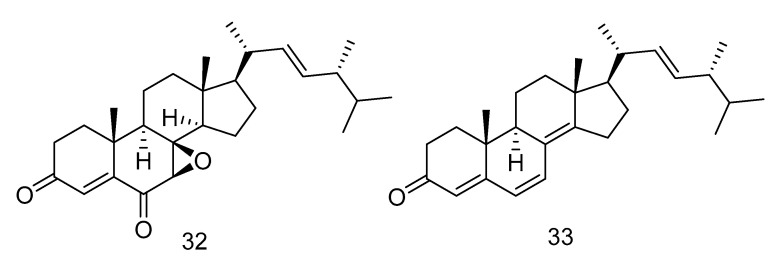
Structures of steroids with antibacterial activity.

7*β*, 8*β*-epoxy-(22E, 24R)-24-methylcholesta-4, 22-diene-3, 6-dione (**32**) and ergosta-4, 6, 8(14), 22-tetraene-3-one (**33**) (Figure 6) were steroids isolated from the extract of *Aspergillus penicillioides* SD-311 from deep sea-sediment collected of the South China Sea. Compound **32** could inhibit *Vibrio anguillarum* with the MIC (minimum inhibitory concentration) value of 32.0 mg/mL. And compound **33** showed antibacterial activity against *Edwardsiella tarda* and *Micrococcus luteus* with MIC values both of 16 mg/mL [23].

**Figure 7:**
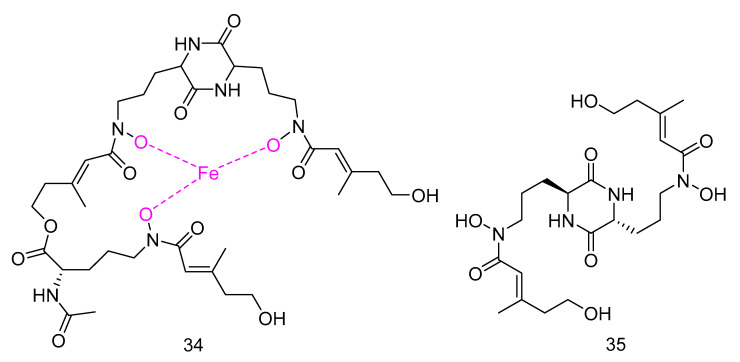
Structures of Mycosphazine A and B.

Mycosphazine A (**34**) was isolated from the extract of *Mycosphaerella* sp. SCSIO z059. It is a new a new iron(III) chelator of coprogen-type siderophore which could greatly promote the biofilm formation of *Bacillus amyloliquefaciens* with the rate of about 249% at concentration of 100 μg·mL^−1^. Its alkaline hydrolysate was a new epimer of dimerum acid, mycosphazine B (**35**) (Figure 7) which showed the same activity with the rate of about 524% at concentration of 100 μg·mL^−1^ [24].

**Figure 8:**
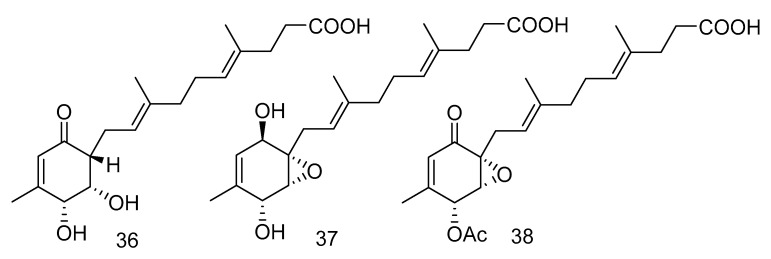
Structures of farnesylcyclohexenones with antibacterial activity.

Peniginsengins C–E (**36**–**38**) (Figure 8) were new farnesylcyclohexenones isolated from the extract of *Penicillium* sp. YPGA11 from the sea water in the Yapu Trench. They showed activity against methicillin-resistant *Staphylococcus aureus* (Methicillin-resistant *Staphylococcus aureus* (MRSA), and anti-methicillin-sensitive *Staphylococcus aureus* (Methicillin-Sensitive *Staphylococcus aureus*, MSSA), with the MICvalues ranging from 8 μg/mL to 64 μg/mL [25].

**Figure 9:**
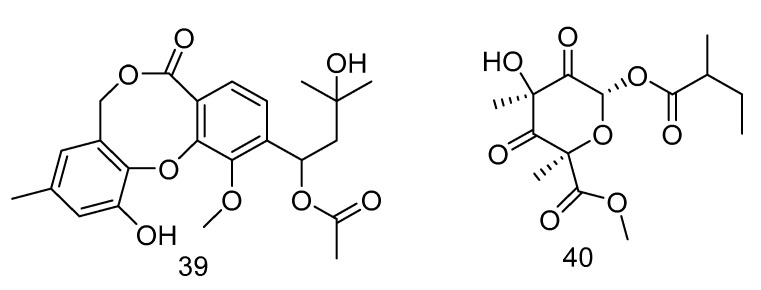
Structures of Canescenin A–B.

Canescenin A–B (**39**–**40**) (Figure 9) were isolated from the extract of *Penicillium canescens* SCSIO z053 derived from the deep-sea sediment of Okinawa Trough. Both of the compounds showed weak antibacterial activities toward *B. amyloliquefaciens* and *P. aeruginosa* at 100 μM [24].

**Figure 10:**
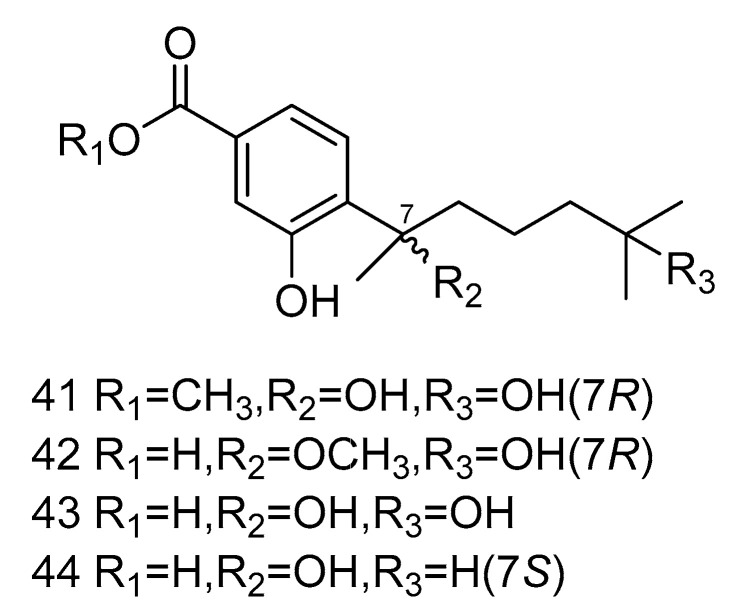
Structures of bisabolane-type sesquiterpenoid with antibacterial activity.

Four bisabolane-type sesquiterpenoid derivatives *ent*-aspergoterpenin *C* (**41**), *7-O*-methylhydroxysydonic acid (**42**), hydroxysydonic acid (**43**) and sydonic acid (**44**) (Figure 10) were isolated from *Aspergillus versicolor* derived from the deep-sea sediment of South China Sea. Compounds **41**–**42** had strong antibacterial activity, whose MIC values against *Escherichia coli*, *Edwardsiella tarda*, *Vibrio harveyi* and *Vibrio parahaemolyticus* were all below or equal to 8.0 µg/mL. Moreover, compound **43** exihibited antibacterial activities against *Aeromonas hydrophilia*, *Escherichia coli*, *Vibrio anguillarum* and *Vibrio harveyi*, with the MIC value of 4.0 µg/mL, equal to the positive control Chloramphenicol [25].

**Figure 11:**
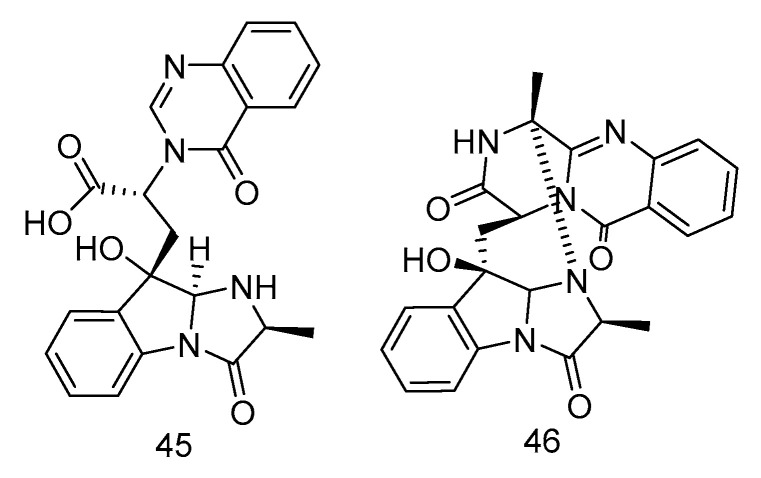
Structures of quinazoline-containing indole alkaloids with antibacterial activity.

Fumigatosides E (**45**) and F (**46**) (Figure 11) were quinazoline-containing indole alkaloids, isolated from the extract of *Aspergillus fumigatus* from the deep-sea sediments of the Indian Ocean. Both of them showed potent antibacterial activities. The MIC values of compound **45** against *Acinetobacter baumannii* ATCC 19606, *Acinetobacter baumannii* ATCC 15122, *Staphylococcus aureus* ATCC 16339 and *Klebsiella pneumoniae* ATCC 14578 were: 12.5 ± 0.042, 6.25 ± 0.035, 6.25 ± 0.13, 12.5 ± 0.098 µg/mL, and the MIC value of compound **46** against *Acinetobacter baumannii* was 6.25 ± 0.033 µg/mL [26].

**Figure 12:**
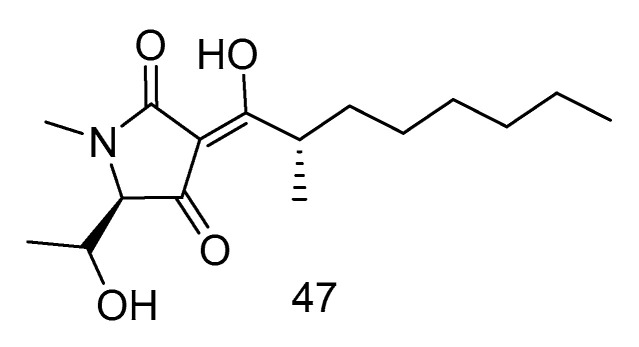
Structure of Penicillenol A2.

Penicillenol A2 (**47**) (Figure 12) was isolated from the extract of *Penicillium biourgeianum* isolated from the sediments of the South China Sea with an inhibitory effect on MSSA. The diameter of inhibitory zone (ZD) is 6.75 ± 0.25 mm. Besides, the synergy of compound **47** with penicillin G sodium (Pen), cefotaxime sodium (Ctx) and oxacillin sodium (Oxa) was studied by plate count and Kirby-Bordisk diffusion method. It was found that, in comparison with the control group, the reduction of bacteria in the experimental group using Pen (10 U mL^−1^), Ctx (15 U mL^−1^) and Oxa (1 U mL^−1^) was less than 1 log ^CFU/mL^. Compared with using compound **30** alone, the reduction of viable bacteria in the experimental group using both the above drugs and compound **47** was greater than or equal to 2 log ^CFU/mL^. Therefore, the combination of compound **30** and β-lactam antibiotics had a synergistic effect, which can increase the sensitivity of MRSA to β-lactam antibiotics [27].

**Figure 13:**
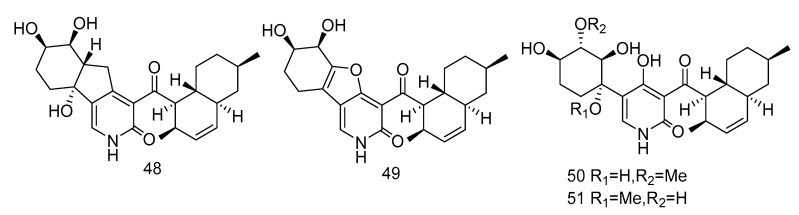
Structures of pyridone alkaloids with antibacterial activity.

Pyridone alkaloids, apiosporamide (**25**) and arthpyrones F–K (**48**–**51**) (Figure 13), were isolated from the extract of *Arthrinium* sp. UJNMF0008 from the sediments of South China Sea, which showed moderate to strong antibacterial activity against *Mycobacterium smegmatis* and *Staphylococcus aureus*, with the IC_50_ values ranging from 1.66–42.8 µM [19].

**Figure 14:**
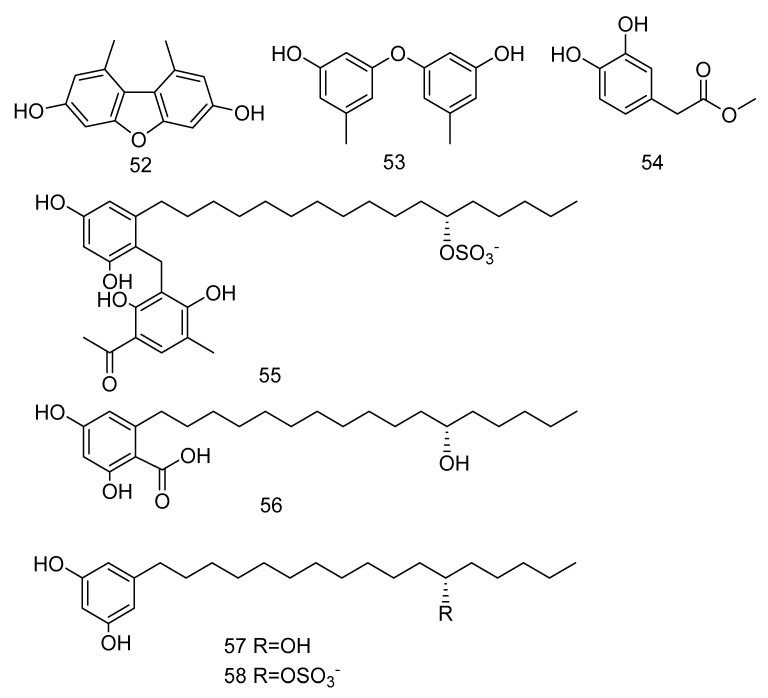
Structures of phenols with antibacterial activity.

3, 5-dimethoxytoluene (**52**), 3, 3′-dihydroxy-5, 5′-dimethyldiphenyl ether (**53**), 3, 4-dihydroxyphenylacetic acid methyl ester (**54**) (Figure 14) were isolated from the extract of *Aspergillus* sp. SCSIO06786 from deep-sea sediments in the Indian Ocean. Compounds **52** and **53** with 50 μg/disc showed inhibition zones against *S. aureus*, MRSA and *E. faecalis*. Compound **54** with 50 μg/disc inhibited the growth of MRSA. In addition, their MIC was tested and the results showed that it was between 3.13–12.5 μg/mL [30]. Penixylarins B–C (**55**–**56**), 1, 3-dihydroxy-5-(12-hydroxyheptadecyl)benzene (**57**), and 1, 3dihydroxy-5-(12-sulfoxyheptadecyl)benzene (**58**) (Figure 14) were isolated from a mixed culture of the Antarctic deep-sea-derived fungus *Penicillium crustosum* PRB-2 with a fungus Xylaria sp. HDN13-249. Compounds **55**–**58** showed activities against *Bacillus tuberculosis*, *B. subtilis* or *Vibrio parahaemolyticus*, MIC values ranging from 6.25 to 100 μM. Among them, the MIC value of compound **56** against *B. tuberculosis* was 6.25 μM, showing the anti-tuberculosis potential [31].

**Figure 15:**
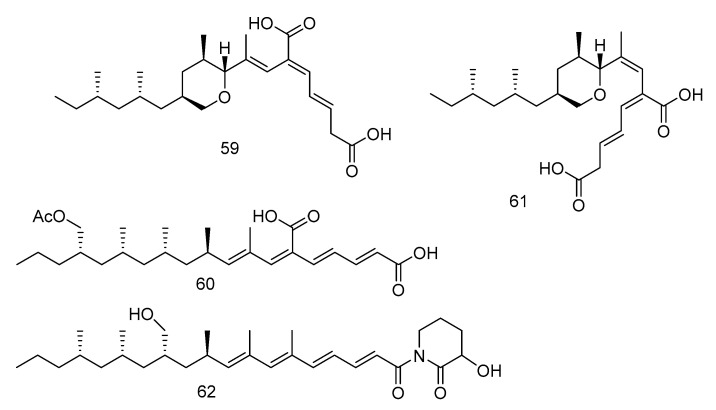
Structures of polypropionate derivatives with antituberculosis activity.

The tyrosine phosphatase (Mptp) secreted by *Mycobacterium tuberculosis* is an important virulence factor of *Mycobacterium tuberculosis* and recognized to be an important target to treat tuberculosis. Tyrosine phosphatase is secreted by *Mycobacterium tuberculosis*, which has two functional phosphatases, PTP A and B (MptpA and MptpB) and enters the cytoplasm of macrophages, preventing the activation of the host’s immune system and regulating the survival of the bacilli in the host [32]. Compounds **59**–**62** (Figure 15) were polyacrylate derivatives with long hydrophobic chains, isolated from the extract of *Aspergillus fischeri* derived from deep sea sediments in Indian Ocean. Compounds **59**–**62** inhibited *M. tuberculosis* protein tyrosine phosphatase B (MptpB) through non-competitive inhibition, with IC_50_ values of 5.1, 12, 4.0 and 11 μM, respectively [33].

#### 2.2.2. Antifungal Secondary Metabolites

**Figure 16:**
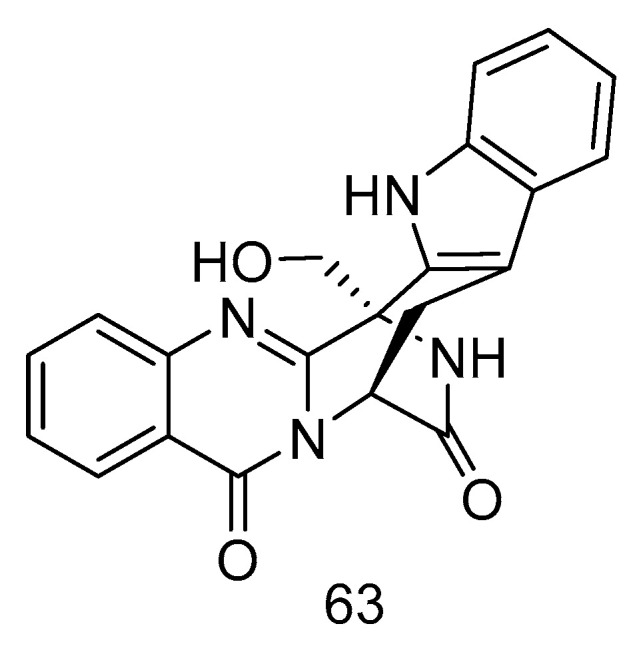
Structure of fumigatoside F.

Quinazoline-containing indole alkaloid, fumigatoside F (**63**) (Figure 16), was isolated from the extract of *Aspergillus fumigatus* derived from deep-sea sediments of the Indian Ocean. The MIC values against *Fusarium oxysporum* f. sp. *cucumerinu* and *Fusarium oxysporum* f. sp. *momordicae* were 25 ± 0.04 and 1.565 ± 0.098 µg/mL, respectively [28].

### 2.3. Secondary Metabolites with Other Bioactivities

In addition to antitumoral and antimicrobial activity, secondary metabolites of deep-sea fungi reported in recent years also have anti-inflammatory and anti-food allergic activities. Compounds **64**–**69** were secondary metabolites from deep-sea fungi with anti-inflammatory activity.

**Figure 17:**
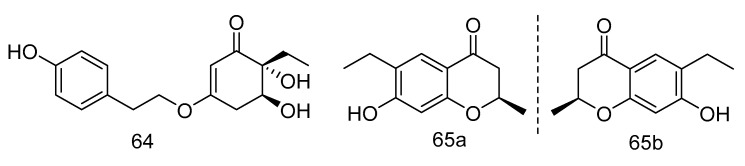
Molecular structures of Trieffusols C and D.

Trieffusols C (**64**) and D (**65**) (Figure 17) were isolated from the extract of *Trichobotrys effuse* from deep-sea sediments of the South China Sea with inhibition of nitric oxide (NO) production in murine macrophages. Their IC_50_ values were 51.9 and 55.9 μM, which is equivalent to the positive control aminoguanidine (IC_50_: 24.8 μM) [34].

**Figure 18:**
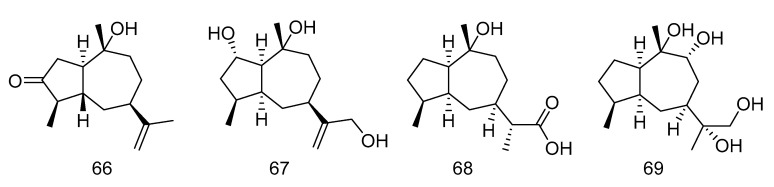
Molecular structures of sesquiterpenoids.

Graphostromane D (**66**), graphostromane F (**67**), graphostromane I (**68**) and (*1R*, *4S*, *5S*, *7S*, *9R*, *10S*, *11R*)-guaiane-9, 10, 11, 12-tetraol (**69**) (Figure 18) were sesquiterpenoids isolated from the extract of *Graphostroma* sp. MCCC 3A00421 derived from hydrothermal sulfide deposit. Compound **50** showed anti-infammatory activity against LPS-induced NO production in RAW264.7 macrophages, with an IC_50_ value of 14.2 μM, even stronger than that of positive contrast. Meanwhile compound **66**, **68** and **69** exhibited weak anti-infammatory activities, with the IC_50_ values of 72.9, 79.1, and 88.2 μM [35].

**Figure 19:**
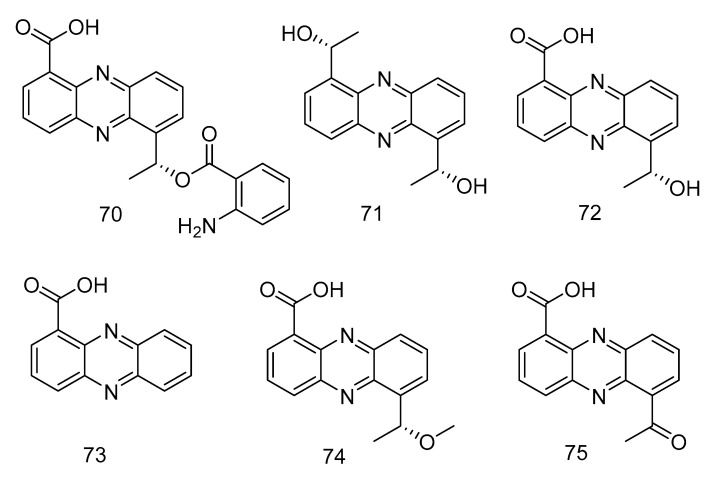
Structures of phenazine derivatives with anti-inflammatory activity.

Phenazine derivatives, 6-[1-(2-aminobenzoyloxy)ethyl]-1-phenazinecarboxylic acid (**70**), saphenol (**71**), (*R*)-saphenic acid (**72**), phenazine-1-carboxylic acid (**73**), 6-(1-hydroxyehtyl)phenazine-1-carboxylic acid (**74**) and 6-acetyl-phenazine-1-carboxylic acid (**75**) (Figure 19), were isolated from the extract of *Cystobasidium laryngis*, which inhibits the NO in mouse macrophage RAW 264.7 cells induced by Lipopolysaccharide (LPS), and does not affect the viability of RAW 264.7 cells at a concentration of up to 30 µg/mL. Compounds **72**, **74**, **75** showed similar inhibitory effects, of which compound **74** had the most obvious inhibitory effect, with the concentration for 50% of maximal effect (EC_50_) value of 19.6 µM. Methylated compound **74** and oxidized compound **75** showed no significant differences in activity mentioned above, but their strength was twice as strong as compound **70** (EC_50_ = 46.8 µM) substituted with 2-aminobenzoic acid. In addition, when there is no functional group substitution at C-6 of phenazine (**73**, EC_50_ = 76.1 μM), the activity is the lowest [36].

The occurrence of food allergic diseases may be related to excessive immune response. Allergens are usually harmless foods such as milk, eggs, fish, peanuts and grains [39]. Acute hypersensitivity is triggered by factors released by mast cells when allergens interact with membrane-bound immune proteins (IgE) [40].

**Figure 20:**
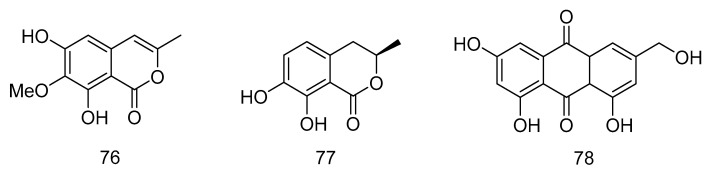
Structures of polyketides with antifood allergic activity.

Polyketides **76**–**78**(Figure 20) were isolated from *Graphostroma* sp. MCCC 3A00421 derived from hydrothermal sulfide, which showed antifood allergic activity. Reticulol (**76**) showed effective inhibition of immunoglobulin E-mediated rat basophilic leukemia-2H3 cells (RBL-2H3) degranulation, with an IC_50_ value of 13.5 µM, which was about seven times stronger than the commercially available anti-food allergy drug loratadine (IC_50_ = 91.6 µM), while 7, 8-dihydroxy -3-methyl-3, 4-dihydroisocoumarin (**77**) and hydroxyemodin (**78**) showed weaker effects, with IC_50_ values of 154.1 and 139.3 µM [37].

**Figure 21:**
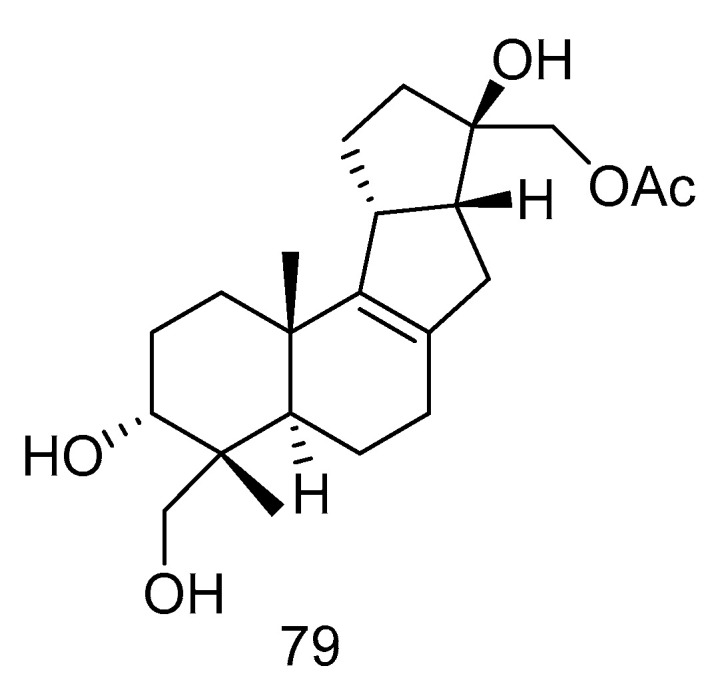
Structure of Botryotin A.

Botryotin A (**79**) (Figure 21) was isolated from *Botryotinia fuckeliana* derived from deep-sea water of the Western Pacific Ocean, which showed moderate antiallergic effect with the IC_50_ value of 0.2 mM [38].

## 3. Secondary Metabolites from Deep-Sea Derived Bacteria

Bacteria from deep-sea sediments are a good source of marine natural products, and their secondary metabolites are usually novel in structure with significant biological activities [41,42,43,44,45]. In particular, actinomycetes are currently proven to be the most important sources of biologically active natural products with clinical or pharmaceutical applications [46]. According to the references, 40 (16 novel) natural products were discovered from deep-sea derived bacteria in the past three years, 19 among which showed biological activities (Table 2).

### 3.1. Antitumoral Secondary Metabolites

Compounds **80**–**87** all showed potent cytotoxic activity.

**Figure 22:**
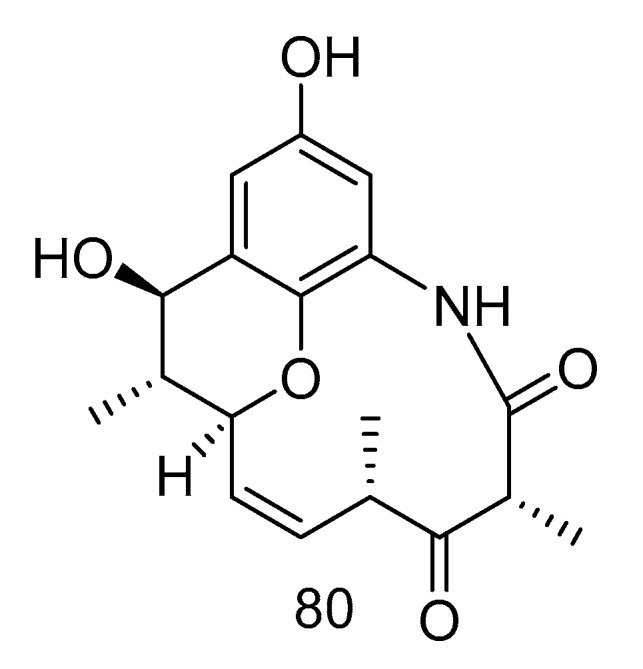
Structure of Cebulactam A2**.**

Cebulactam A2 (**80**) (Figure 22) was a polyketide isolated from the extract of *Saccharopolyspora cebuensis* derived from Atlantic deep-sea sediments, which had a weak antiproliferative effect on human cervical cancer cell Hela and human lung cancer cell H1299, the inhibition rates (20.00 μg/mL) were 35.0 and 31.0%, respectively [47].

**Figure 23:**
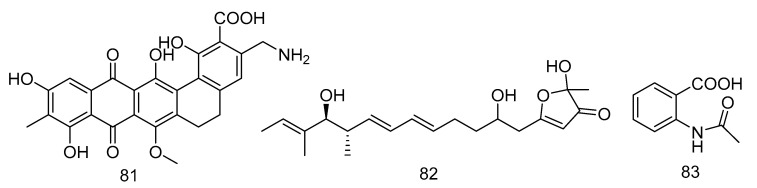
Structures of aromatic polyketides with antitumor activity.

Akazamicin (**81**), actinofuranone C (**82**) and N-formylanthranilic acid (**83**) (Figure 23) were isolated from the extract of *Nonomuraea* sp. AKA32 derived from seawater of Sagami Bay in Japan. Compounds **81** and **82** showed the same level of cytotoxic activity against B16 melanoma cells, with IC_50_ values of 1.7 and 1.2 µM, respectively. Compound **83** showed about 10 times lower cytotoxic activity than that of compounds **81** and **82**. The cytotoxic activity of these three compounds against human liver cancer cells Hep G2 and human colorectal adenocarcinoma cells Caco-2 was not obvious, with IC_50_ values ranging from 10 to 200 μM [48].

**Figure 24:**
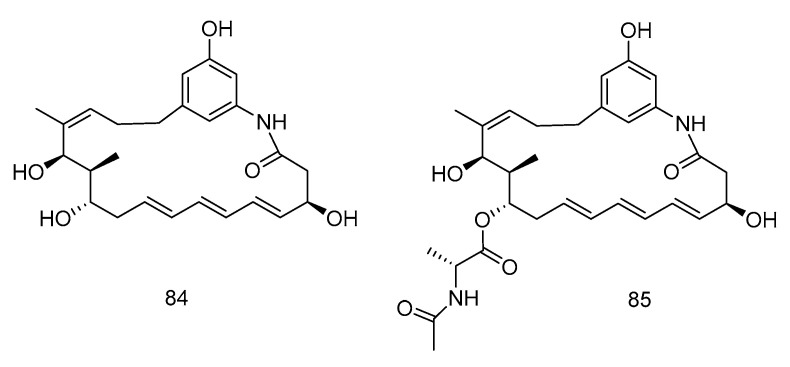
Structures of Ansamycins with antitumor activity.

**Figure 25:**
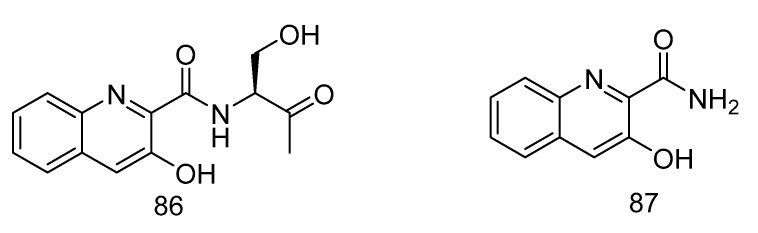
Structures of (*S*)-3-hydroxy-N-(1-hydroxy-3-oxobutan-2-yl) quinoline-2-carboxamide and 3-hydroxyquinoline-2-carboxamide.

Trienomycins J–H (**84**–**85**) (Figure 24) were isolated from the extract of *Ochrobactrum* sp. OUCMDZ-2164 derived from deep-sea water of the South China Sea. Compound **84** exhibited antitumor activity against human breast cancer cells (MCF-7) with 61.5% inhibition rate at 10 μmol/L [49]. Compound **85** showed cytotoxic activity against human lung carcinoma cell line (A549) and human leukemia cell line (K562) with IC_50_ values of 15 and 23 µM, respectively [50].

(*S*)-3-hydroxy-N-(1-hydroxy-3-oxobutan-2-yl) quinoline-2-carboxamide (**86**) and 3-hydroxyquinoline-2-carboxamide (**87**) (Figure 25), were isolated from a solitary coral derived *Streptomyces cyaneofuscatus* from Biscay Bay of north Atlantic. The IC_50_ values towards human liver cancer cell HepG2 were 15.6 and 51.5 µM, respectively [51].

### 3.2. Antimicrobial Secondary Metabolites

Compounds **88**–**96** all showed potent antibacterial activity.

Aborycin (**88**) was a lasso peptide isolated and identified from the deep-sea-derived microbe *Streptomyces* sp. SCSIO ZS0098 which was isolated from the deep-sea sediments of the South China Sea. Shao et al. [52] identified the aborycin biosynthetic gene cluster (abo) on the basis of genomic sequence analysis, and then heterologously expressed in *Streptomyces coelicolor* to obtain compound **86**. The compound had moderate bacteriostatic activity against 13 *Staphylococcus aureus* strains from various sources, with MIC values between 8.0–128 µg/mL. The MIC values of compound **88** against *Enterococcus faecalis* and *Bacillus thuringiensis* were 8.0 µg/mL and, 2.0 µg/mL, respectively. In addition, compound **88** had significant antibacterial activity against the poultry pathogen *Enterococcus enterococci* (MIC = 0.5 µg/mL) [44]. Atratumycin (**89**) was also a peptide isolated from the extract of *Streptomyces atratus* from deep-sea sediments of the South China Sea, which is a cyclic dipeptide that has activity against *Mycobacterium tuberculosis*, whose MIC values were 3.8 and 14.6 µM against *M. tuberculosis* H37Ra and H37Rv [53].

**Figure 26:**
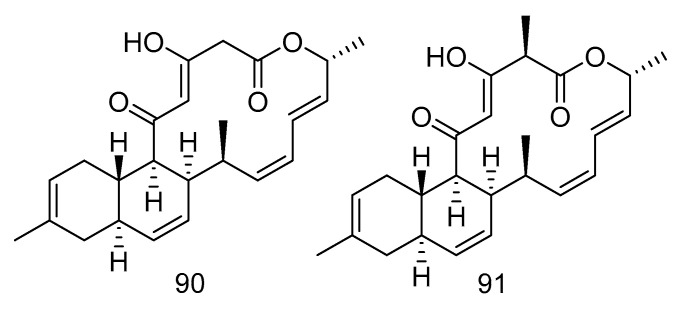
Structures of anthracimycin B and anthracimycin.

Compounds **90**–**94** are all polyketides. Anthracimycin B (**90**) and anthracimycin (**91**) (Figure 26) were isolated from the extract of *Streptomyces cyaneofuscatus* isolated from a gorgonian coral collected in the 1500 m Avilis submarine canyon. They were sensitive to Gram-positive pathogens MRSA, MSSA, vancomycin-sensitive *Enterococcus faecium* and vancomycin-sensitive *Enterococcus faecalis* and all showed strong antibacterial effects. The MIC value of compound **90** was less than 0.03 µg/mL, and the MIC value of compound **91** was between 0.125–8 µg/mL. Compound **90** also had anti-tuberculosis activity, with the MIC value of 1–2 µg/mL [54].

**Figure 27:**
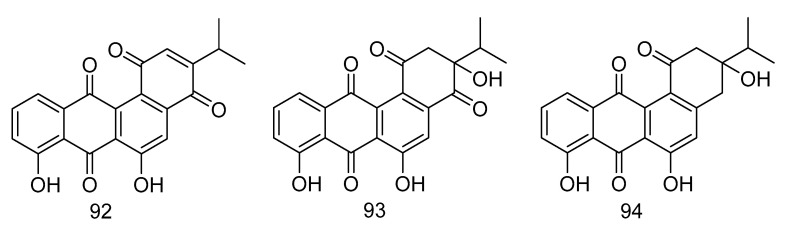
Structures of nocardiopsistins A–C.

Nocardiopsistins A–C (**92**–**94**) (Figure 27) were isolated from the extract of *Nocardiopsis* sp. HB-J378 isolated from a deep-sea sponge *Theonella* sp. Compound **93** had the same MIC (3.12 µg/mL) as the positive control chloramphenicol, while compounds **92** and **94** had moderate anti-MRSA activity (MIC = 12.5 µg/mL) [55].

**Figure 28:**
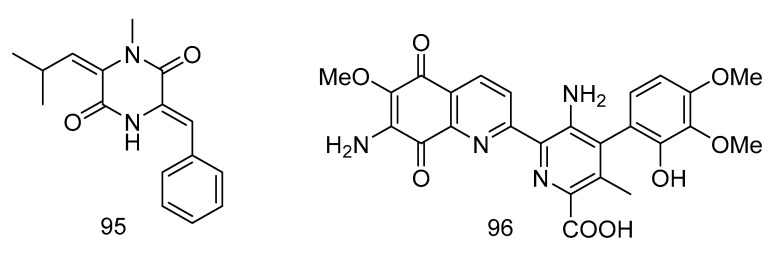
Structures of 1-*N*-methyl- (*E*, *Z*)-albonoursin and streptonigrin.

1-*N*-methyl-(*E, Z*)-albonoursin (**95**) and streptonigrin (**96**) (Figure 28) were alkaloids isolated from the extract of *Streptomycetes* sp. strain SMS636 from deep-sea sediments in the South China Sea. Compound **95** showed moderate antibacterial activity towards *Staphylococcus aureus* and MRSA, with MIC values of 12.5 and 25 µg/mL, respectively. The MIC value of compound **96** was 0.78 µg/mL for *Staphylococcus aureus* and MRSA, and had anti-BCG (Bacillus Calmette-Guérin, BCG) activity with a MIC value of 1.25 µg/mL [56].

### 3.3. Other Bioactive Secondary Metabolites

**Figure 29:**
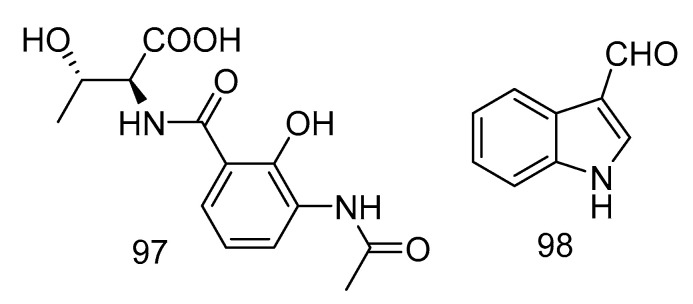
Structures of acantimycic acid and Indol-3-carbaldehyde.

Acantimycic acid (**97**) was an alkaloid with good neuroprotection. It was isolated from the extract of *Alcanivorax* sp. SHA4 from deep-sea sediments of the Western Pacific and could inhibit the cell damage caused by glutamic acid to PC12 cells. The protective effect was more obvious at low concentration [57]. Indol-3-carbaldehyde (**98**) was isolated from the extract of *Saccharopolyspora cebuensis* derived from Atlantic deep-sea sediments. It showed weak anti-allergic effect with the IC_50_ value of 55.75 μg/mL [47]. The structures of compounds **97** and **98** are shown in Figure 29.

## 4. Research Methods for Diversity of Secondary Metabolites from Deep-Sea Microorganisms

### 4.1. Isolation and Cultivation of Deep-Sea Microorganisms

Marine microorganisms have the following characteristics: (1) can grow and/or form spores in the marine environment; (2) form a symbiotic relationship with other marine organisms; or (3) adapt and evolve at the genetic level or have metabolic activity in the marine environment [58]. It is estimated that the diversity of marine fungi exceeds 10,000 species [59,60], but so far only about 1250 species have been described [61,62]. However, deep-sea microbial research starts late for its difficulties in collection and cultivation, so people face more challenges in the exploration of its secondary metabolites.

#### 4.1.1. Sample Pretreatment

In natural samples without pretreatment, the isolation frequency of bacteria is higher than that of fungi [63]. Different pretreatment methods should be adopted for different target strains to improve the isolation efficiency.

Because the actinomycete spores have a certain heat resistance, dry and wet heat treatment can effectively reduce other bacterial contamination [64,65,66,67,68]. Dry heat treatment can inactivate bacteria, and at the same time induce the germination of actinomycetes spores to a certain extent; the principle of wet heat treatment is to denature and inactivate non-target strain proteins in the sample by heating in a water bath. In addition to heat treatment, the commonly used pretreatment methods include chemical reagent treatment [69], differential centrifugation [70] and so on.

Microwave treatment can not only significantly increase the number of isolated alkaliphilic and halophilic marine actinomycetes, but also significantly increase the isolation of rare marine actinomycetes. Ding et al. [71] used 120 W, 2450 MHz microwave and ice-water mixture to process one part of the suspension in the treatment of sea mud samples. After gradient dilution, they were applied to three separate media. In the seven samples after microwave treatment, the number of rare alkaliphilic marine actinomycetes in four samples and the halophilic marine actinomycetes in three samples increased significantly. Therefore, microwave processing also has certain application value.

#### 4.1.2. Medium Selection and Improvement

When the strains are separated, the medium as a nutrient source plays an important role in the growth and metabolism of the strains. Different culture media provide different carbon and nitrogen sources for different microorganisms to grow, so it is necessary to select the appropriate culture media for microorganism screening.

For fungi, we usually use common media such as: Potato Dextrose Agar Medium (PDA), Czapek Dox Agar Medium (CDA), Sabouraud Dextrose Agar Medium (SDA), Corn Meal Agar Medium (CMA), Malt Extract Agar Medium (MEA), yeast malt agar medium (YM), etc. He et al. [72] used the above-mentioned six media (all added chloramphenicol and streptomycin sulfate to inhibit growth of bacteria) to separate samples from the deep-sea sediment samples of Yapu Trench. In their study, YM media was the best from the perspective of the isolation ability of six different media, which obtained nine kinds of fungi; followed by PDA which allowed the retrieval of eight different fungal species. The worst were CMA (three kinds) and CDA (two kinds).

For bacteria, according to the main components of the medium, it can be divided into marine agar medium (MA), synthetic medium for selective isolation of actinomycetes (Actinomycete Isolation Agar, AIA), starch medium, natural ingredient medium, high salt medium and other media (Table 3). Chen et al. [73] used the 23 media in the table to isolate bacteria in the 4000 m deep-sea sediments of the South China Sea, and most of natural products from the strains obtained from the deep sea sediment environment, are antibiotic, cytotoxins, with high efficiency enzyme activity, and tolerant for unfavorable environment, degradation of refractory pollutants and other characteristics suitable for the unique marine extreme environment.

### 4.2. Screening Methods of Deep-Sea Natural Products

One of the keys to develop and utilize biological resources is how to obtain bioactive natural products from cultivable deep-sea microorganisms. Traditional natural product activity screening method is also suitable for the activity screening of deep-sea microbial metabolites, which mainly tracks the active substances in the cultivation broth. In addition, commonly used methods include model screening for specific target modeling and evaluation, and gene screening based on microbial natural product synthesis gene clusters.

#### 4.2.1. In Vivo Screening Methods

In vivo screening models mainly refer to animal models and Serum pharmacology models. Animal models can mimic clinical features such as physiology and pathology similar to those of patients. Serum pharmacology models can help prove the true positive compounds, whether they are original drugs or metabolites [74]. Therefore, in vivo experiments have an irreplaceable role in activity screening. However, due to its time-consuming, low throughput, and large sample consumption, it is less used in preliminary screening.

#### 4.2.2. Cell and Receptor/Enzyme Model Screening Methods

Cell and receptor/enzyme model screening is used for target screening, and is usually established as a specific and effective model on pharmacology at the cellular or molecular level.

Compared with simple chemical methods, evaluation of biological activity of natural products based on cell models can not only simulate the human physiological environment, but it can also explore and evaluate the biological activity and mechanism of natural products at multiple targets; compared with animal experiments in vivo, it not only shorten the experimental time, but greatly reduces the experimental cost [75]. The most commonly used cell models are human cancer cell lines, such as: A54, MCF7, HepG2, Caov-3, PANC-1 and so on. In addition, there are other models at the cellular level to test other bioactivities. Xu et al. used hemolysis assay on sheep red blood cell to test the anti-complement activity of 42 strains of marine actinomycetes isolated from Dalian Xinghai Bay mud samples, and further isolated three small molecular compounds with weak anti-complement activity from extract of strain DUT11 [76].

Receptors or enzymes related to various physiological and pathological processes in the body are considered to be one of the main targets of drug action [74]. Liu et al. tested IC_50_ values of the polypropionate derivatives against MptpB to show their antituberculosis activities [33]. ACE2 has been shown to be the main receptor for SARS-CoV S protein to infect cells [77]. Deng et al. showed that baicalin had an inhibitory activity against ACE with the IC_50_ value of 2.24 mM [78].

#### 4.2.3. Virtual and Gene Screening Methods

Virtual screening based on compound structural diversity, that is, using computer programs to screen bioactive compounds from existing virtual libraries. And compounds with higher chemical structure spatial diversity are more suitable for virtual library establishment [79].

Gene screening breaks through the traditional active screening model. Because the secondary metabolite synthetic gene clusters with similar structures have a certain degree of similarity, the strains that produce the target compound can be obtained from nature by screening specific gene clusters.

Polyketide compounds are catalyzed by a type of polyketide synthetase (PKS) which is widely present in nature. Polyketide synthetase can generally be divided into three types according to its protein structure and catalytic mechanism, namely type I, type II and type III [80]. Type I PKS includes type I modular PKS (bacteria) and type I repeat PKS (fungi). A typical type I module PKS is a multifunctional complex enzyme composed of modules. Each module contains a unique and non-repetitive structural domain, which mainly contains acyltransferase (acyltransferase, AT), β-keto synthase (ketosynthase, KS) responsible for catalyzing the formation of carbon-carbon bonds and extending the main chain, and acyltransferase cylcar-rier protein (acyltransferase cylcar-rier protein, ACP) responsible for receiving and transporting acyl units provided by the AT domain), these three domains constitute the smallest catalytic module and are also the three essential domains of PKS.

Non-ribosomal peptide synthetase (NRPS) is also widely present in bacteria, fungi and plants, and uses different amino acids as substrates to catalyze the production of condensed peptides. NRPS is mainly composed of different independent modules. Each module contains an adenylation structural functional domain (andeylation, A) that selects and activates special amino acids, and loads aminoacyl residues into the sulfhydryl structural functional domain (thiolation, T), and the condensation domain (C) of peptide compounds that polymerize activated amino acids to produce amides.

In fungi, the genes encoding PKS and NRPS can be aggregated to produce type I repeat PKS units (KS, AT, DH, CMeT, KR and ACP domains) and NRPS units (A, T and C domains) PKS-NRPS hybrid enzyme. PKS-NRPS has the function of catalyzing the combination of PKS products and NRPS products, thereby producing more abundant natural products-PKS-NRPS hybrid compounds [81]. Such PKS-NRPS hybrid compounds are often a class of natural products with complex and diverse structures and a wide range of biological activities. They not only play an important role in the survival and prosperity of the host in the natural environment, but also is an important source for the discovery of active lead compounds with potential applications.

Jiang et al. [82] applied the type I polyketide synthase (PKS-I) gene screening system and DNA sequence similarity comparison to select positive *Actinoplanes* sp. from 32 strains of marine actinomycetes. *Actinoplanes* sp. FIM060065 was one of them. And from its fermentation broth researchers obtained a macrolide compound homogenous to tiacumicin B by High Performance Liquid Chromatography (HPLC) preparation, which showed strong antibacterial activity towards Gram-positive bacteria, such as *Clostridium difficile*, *Streptococcus pneumoniae*, *Bifidobacterium*, etc. Vanessa Rédou et al. [83] isolated 124 filamentous fungi and 59 yeasts from sediments in the Canterbury basin of New Zealand. The PKS-NRPS analysis results showed that there was no PKS-NRPS hybrid gene in the yeast genome; compared with yeast, filamentous fungal isolates from deep seabed sediments have greater bioactive compound synthesis potential, but they have fewer bioactive compound genes than those isolated from shallower depths.

### 4.3. Secondary Metabolite Discovery Based on Synthetic Biology

Synthetic biology is a multidisciplinary disruptive study leading a new generation of biotechnology revolution, in which gene editing takes an important part. Gene editing has unique advantages in establishing an artificially regulated biosynthetic system, further mining new natural product resources of actinomycetes, solving the bottleneck of existing natural products and developing derivatives. Recently, CRISPR (clustered regularly interspaced short palindromic repeats)/Cas9 system, known as “magic scissor”, was found to improve the efficiency of gene editing.

Elizabeth J. Culp et al. [84] applied CRISPR/Cas9 system to 11 actinomycete strains, knocked out the common streptomycin and streptomycin genes, produced a variety of hidden rare antibiotics, and constructed a platform that can be widely used to stimulate the potential of microbial secondary metabolism. Indra Roux et al. [85] established the first CRISPRa system for filamentous fungi and discovered the *mic* cluster product, dehydromicroperfuranone. Meanwhile, factors affecting the efficiency of the system was also studied.

## 5. Conclusions and Perspective

Although the research on secondary metabolites of deep-sea microbes started later than that in other environments [9], it has drawn much more attention, and natural products with novel structures and good biological activities have been discovered in the past three years. From our literature review, fungi seem to be the focus of most isolations from the deep-sea for bioprospection of metabolites with biological activities; also, producers of higher diversity and amount of compounds. Among bacteria, actinomycetes seem to be studied more deeply in natural product research, and they have shown the potential to become biological resources with novel structures and good biological activities. When it comes to structural classes, polyketides showed a broad spectrum of bioactivities, such as antitumor, antibacterial, anti-inflammatory and antifood allergic.

The rapid development of deep sea exploration and bioinformatics has provided solid technical support for the chemical diversity and bioactivity diversity of secondary metabolites of deep sea microorganisms, but there are still many challenges, such as activation of specific biosynthetic gene clusters and heterologous expression, directed transformation of synthetic gene clusters, design of virtual screening libraries for natural products, and how to solve the problem of yield of active compounds.

Deep-sea microbial natural product resources are still a virgin land that needs to be developed urgently. Reasonable and green applied research will contribute more power to drug discovery.

## Figures and Tables

**Table 1:** Bioactive Natural products from deep-sea derived fungi in 2018–2020.

Bioactivity	Fungal Species	Structural Class	Number	Depth (m)	Region	Source	Reference
antiallergic	*Botryotinia fuckeliana*	diterpenoid	79	5572	Western Pacific Ocean	sea water	[38]
antibacterial	*Aspergillus penicillioides*	steroid	32, 33	2038	South China Sea	sediment	[23]
antibacterial	*Mycosphaerella* sp. SCSIO z059	iron (III) chelator	34	1330	Okinawa Trough	sediment	[24]
antibacterial	*Mycosphaerella* sp. SCSIO z059	dimerum acid	35	1330	Okinawa Trough	sediment	[24]
antibacterial	*Penicillium* sp. YPGA11	polyketide	36–38	4500	West Pacific	sea water	[25]
antibacterial	*Penicillium canescens* SCSIO z053.	polyketide	39, 40	1387	Okinawa Trough	sediment	[26]
antibacterial	*Aspergillus versicolor*	sesquiterpene	41–44	1487	South China Sea	sediment	[27]
antibacterial	*Aspergillus fumigatus*	alkaloid	45, 46	3614	India Ocean	sediment	[28]
antibacterial	*Penicillium biourgeianum*	alkaloid	47	2226	South China Sea	sediment	[29]
antibacterial	*Arthrinium* sp. UJNMF0008	alkaloid	25, 48–51	3858	South China Sea	sediment	[19]
antibacterial	*Aspergillus* sp. SCSIO06786	phenol	52–56	4762	India Ocean	sediment	[30]
antibacterial	Penicillium crustosum *	phenol	55–58	526	Prydz Bay	sediment	[31]
antifood allergic	*Graphostroma* sp. MCCC 3A00421	polyketide	76–78	2721	Atlantic	hydrothermal sulfide	[37]
antifungal	*Aspergillus fumigatus*	alkaloid	63	3614	India Ocean	sediment	[28]
anti-inflammatory	*Trichobotrys effuse*	polyketide	64, 65	1428	South China Sea	sediment	[34]
anti-inflammatory	*Graphostroma* sp. MCCC 3A00421	sesquiterpenoid	66–69	2721	Atlantic	hydrothermal sulfide	[35]
anti-inflammatory	*Cystobasidium laryngis*	alkaloid	70–75	4317	India Ocean	sediment	[36]
antituberculosis	*Aspergillus fischeri*	polyketide	60–63	3000	India Ocean	sediment	[33]
cytotoxic	*Penicillum citreonigrum*	polyketide	1, 2	2910	Southeast India Ocean	sediment	[11]
cytotoxic	*Phomopsis lithocarpus*	polyketide	3, 4	3606	India Ocean	sediment	[12]
cytotoxic	*Chaetomium globosum*	polyketide	5, 6	2476	South China Sea	sediment	[13]
cytotoxic	*Penicillium chrysogenum*	polyketide	7–15	2076	South Atlantic Ocean	sediment	[14]
cytotoxic	*Hypoxylon rubiginosum*	polyketide	16–19	4188	South China Sea	sediment	[15]
cytotoxic	*Penicillium griseofulvum*	sesquiterpene	20	1420	India Ocean	sediment	[16]
cytotoxic	*Botryotinia fuckeliana*	diterpene	21	5572	West Pacific	sea water	[17]
cytotoxic	*Phomopsis tersa*	meroterpenoid	22, 23	3000	India Ocean	sediment	[18]
cytotoxic	*Chaetomium globosum*	alkaloid	24	2476	South China Sea	sediment	[13]
cytotoxic	*Arthrinium* sp. UJNMF0008	alkaloid	25	3858	South China Sea	sediment	[19]
cytotoxic	*Cladosporium sphaerospermum*	alkaloid	26	4571	East India Ocean	sediment	[20]
cytotoxic	*Cladosporium sphaerospermum*	alkaloid	27–29	6562	Mariana Trench	sediment	[21]
cytotoxic	*Sarcopodium* sp. FKJ-0025	phenol	30, 31	200	Kagoshima coast	sediment	[22]

* refers to a mixed culture of the Antarctic deep-sea-derived fungus *Penicillium crustosum* PRB-2 and the mangrove-derived fungus *Xylaria* sp. HDN13-249.

**Table 2:** Bioactive Natural products from deep-sea derived bacteria in 2018–2020.

Bioactivity	Bacterial Species	STRUCTRUAL CLASS	Number	Depth (m)	Region	Source	Reference
anti-allergic effect	*Saccharopolyspora cebuensis*	polyketide	98	2875	Atlantic	sediment	[47]
cytotoxic	*Saccharopolyspora cebuensis*	polyketide	80	2875	Atlantic	sediment	[47]
cytotoxic	*Nonomuraea* sp. AKA32	polyketide	81–83	800	Sagami Bay	sea water	[48]
cytotoxic	*Ochrobactrum* sp. OUCMDZ-2164	polyketide	84	2000	South China Sea	sea water	[49]
cytotoxic	*Ochrobactrum* sp. OUCMDZ-2164	polyketide	85	2000	South China Sea	sea water	[50]
cytotoxic	*Streptomyces cyaneofuscatus*	alkaloid	86, 87	2000	Biscay Bay	solitary coral	[51]
antibacterial	*Streptomyces* sp. SCSIO ZS0098	peptide	88	3000	South China Sea	sediment	[52]
antibacterial	*Streptomyces atratus*	peptide	89	3536	South China Sea	sediment	[53]
antibacterial	*Streptomyces cyaneofuscatus*	polyketide	90, 91	1500	Avilés submarine Canyon	gorgonian coral	[54]
antibacterial	*Nocardiopsis* sp. HB-J378	polyketide	92–94	——	——	*Theonella* sp.	[55]
antibacterial	*Streptomycetes* sp. strain SMS636.	alkaloid	95, 96	3000	South China Sea	sediment	[56]
anti-BCG	*Streptomycetes* sp. strain SMS636.	alkaloid	96	3000	South China Sea	sediment	[56]
inhibit the cell damage	*Alcanivorax* sp. SHA4	alkaloid	97	5180	West Atlantic	sediment	[57]

**Table 3:** Different kinds of medium and their components.

Type of Medium	Name of Medium	Composition of Medium
marine agar medium (MA)	MA; BD Difco™	① marine bacteria medium2216E
	MAB	② 50%MA medium
	MAE	③ 20%MA medium
	MAJ	④ 10%MA medium
Actinomycete Isolation Agar (AIA)	AIA; BD Difco™	① selective medium for actinomycetes
	AIAB	② 50%AIA medium
	AIAE	③ 20%AIA medium
natural ingredient medium	acid microbial medium (AM)	① MgSO_4_ 7 H_2_O 0.50 g, (NH_4_)_2_SO_4_ 0.40 g, K_2_HPO_4_ 0.20 g, KCl 0.10 g, FeSO_4_·7H_2_O 0.01 g, yeast extract 0.25 g
	Maltose-Yeast-Peptone Medium (MYP)	② maltose extract 5.0 g, yeast extract 5.0 g, peptone 5.0 g, NaCl 3.0 g
	nutrient medium^®^	③ peptone 10.0 g, yeast extract 5.0 g, maltose extract 5.0 g, casein amino acid 5.0 g, beef extract 2.0 g, glycerin 2.0 g, Tween 80 50.0 mg, MgSO_4_·7H_2_O 1.0 g
	R2AB; BD Difco™	④ 50%R2A medium
	R2AJ	⑤ 5%R2A medium
starch medium	MA starch medium (MAS)	① MA, 1% (*m*/*V*) soluble starch
	50%MA starch medium (MABS)	② MAB, 1% (*m*/*V*) soluble starch
	20%MA starch medium (MAES)	③ MAE, 1% (*m*/*V*) soluble starch
	10%MA starch medium (MAJS)	④ MAJ, 1% (*m*/*V*) soluble starch
	5%MA starch medium (MATS)	⑤ 5% MA, 1% (*m*/*V*) soluble starch
high-salinity medium	high-salinity AIA medium (AIAS)	① AIA 10.0 g, NaCl 100.0 g, crude salt extract5.0 g, SrCl_2_ 2.0 g
	high-salinity beef extract medium (BFSM)	② beef extract 2.0 g, CaCO_3_ 1.0 g, crude salt extract 5.0 g, Na_2_MoO_4_ 5.0 g, soluble starch 2.0 g, NaCl 100.0 g
	high-salinity casein medium (CAAM)	③ casein hydrolysate 1.0 g, KCl 2.0 g, MgSO_4_·7H_2_O 2.0 g, NaCl 100.0 g, crude salt extract 10.0 g, gluconate 1.0 g; trisodium citrate1.0 g, yeast extract 1.0 g, KMnO_4_ 2.0 g (sterilize alone)
	high-salinity iron-containing medium (YJSF)	④ MA 15.0 g, CaCO_3_ 5.0 g, NaCl 100.0 g, FeCl_2_ 0.5 g (filter sterilization), FeSO_4_ 0.5 g (filter sterilization)
other medium	SN	① NaNO_3_ 0.75 g, K_2_HPO_4_ 0.0159 g, EDTA-2Na 0.0056 g, Na_2_CO_3_ 0.0104 g, 50% sea water, Vitamin B12 0.001 g(filter sterilization), cyano trace metal solution 1 × 10^−6^ sterilize alone (acetic acid 6.25 g, ammonium ferric citrate 6.0 g, MnCl_2_·4H_2_O 1.4 g, Na_2_MoO_4_·2H_2_O 0.39 g, Co(NO_3_) _2_·6H_2_O 0.025 g, ZnSO_3_·7H_2_O 0.222 g)
	ZANT	② NaHCO_3_ 2.0 g, NaH_2_PO_4_·2H_2_O 0.05 g, NaNO_3_ 0.5 g, CaCl_2_ 0.02 g, MgSO_4_·7H_2_O 0.05 g, KCl 0.1 g, A5 solution 1 × 10^−6^(H_3_BO_3_ 2.86 g, MnCl·4H_2_O 1.80 g, ZnSO_4_·7H_2_O 0.22 g, Na_2_MoO_4_·2H_2_O 0.3 g, CuSO_4_·5H_2_O 0.08 g)
